# Mechanical stress effects on transcriptional regulation of genes encoding microtubule- and actin-associated proteins

**DOI:** 10.1007/s12298-021-01123-x

**Published:** 2022-01-21

**Authors:** Galina V. Shevchenko, Konstantin V. Krutovsky

**Affiliations:** 1 Institute of Botany, National Academy of Sciences of Ukraine, Kiev, 01004 Ukraine; 2 Department of Forest Genetics and Forest Tree Breeding, Georg-August University of Göttingen, Büsgenweg 2, 37077 Göttingen, Germany; 3 Center for Integrated Breeding Research, Georg-August University of Göttingen, 37075 Göttingen, Germany; 4 Laboratory of Population Genetics, N.I. Vavilov Institute of General Genetics, Russian Academy of Sciences, 119333 Moscow, Russian Federation; 5 Department of Genomics and Bioinformatics, Laboratory of Forest Genomics, Genome Research and Education Center, Institute of Fundamental Biology and Biotechnology, Siberian Federal University, 660036 Krasnoyarsk, Russian Federation; 6 Scientific and Methodological Center, G. F. Morozov Voronezh State University of Forestry and Technologies, 394087 Voronezh, Russian Federation

**Keywords:** *Arabidopsis*, Microtubules, Actin filaments, Genes encoding tubulin- and actin-associated proteins, Mechanical stress, Slow rotating clinostats

## Abstract

Plant cytoskeleton regulation has been studied using a new approach based on both (1) pharmacological analysis of tubulin and actin inhibitors and (2) mechanical stimulation achieved by using a slow-rotating (2 rpm) clinostat in combination with transcriptional analysis of genes encoding TUA6, ACT2, MAP65-1, CLASP, PLDδ, FH4 and FH1 proteins in *Arabidopsis thaliana* seedling roots. The obtained data suggest feedback between the organization of microtubule (MT) and actin filament (AF) networks and the expression of the *ACT2, TUA6, MAP65-1, CLASP* and *FH1/FH4* genes. Different regulation of feedback between MT/AF organization and *TUA6, ACT2, MAP65-1, CLASP, FH4* and *FH1* gene expression was noted during slow clinorotation, possibly due to altered mechanical impact on the cortical cytoskeleton. For the first time, the expression of the tubulin-associated gene *MAP65-1* was shown to be dependent upon the organization of AFs. TUA6, MAP65-1, CLASP, FH1 and FH4 likely participate in mechanical signal transduction. Our work demonstrated that slow clinorotation is able to cause mechanical stress.

## Introduction

In interphase plant cells, arrays of cortical microtubules (cMTs), which are composed of microtubule (MT) bundles and separate MTs, are located beneath the plasma membrane (PM) and arranged transversely to the cell axis. They are highly dynamic structures able to reorganize fast upon receiving internal or external stimuli (Yuan et al. 1994). Cortical MTs coalign with actin filaments (AFs), and both MTs and AFs share common functions, including cell division and elongation (Collings 2008; Sampathkumar et al. 2011). In the cortical cell area, AFs and MTs are associated with PM proteins and create the so-called cell wall–PM–cytoskeleton continuum (Wyatt and Carpita 1993; Baluška et al. 2003). Since the cortical cell area is the first to respond to environmental stimuli, the cell wall–PM–cytoskeleton continuum is considered to be a susceptive structure, where environmental stimuli are perceived and transduced. CMTs respond to environmental stresses by reorganization of their patterns, which could impact the cell growth rate. Therefore, the mechanisms regulating MT functioning are of special interest. The high sensory ability of MTs depends on the dynamic instability of the tubulin polymer, making it a good target for mechanical stimulation. Rigid MTs together with flexible actin filaments and the anisotropic cell wall are suggested to form a tensegral system allowing plant cells to sense mechanical strains and minimize the load (Nick 2013). In addition, the plant cytoskeleton has been suggested to respond directly to mechanical stress (Hamant et al. 2019).

The cortical cytoskeleton is a complex structure, and new approaches and designs of new experiments are required to reveal the details of its regulation. We consider investigation of cytoskeleton arrangements during clinorotation to be a promising approach to this type of research. Slow clinorotation which disorients plants in relation to gravity vector is applied widely for modeling microgravity conditions on Earth (Kiss et al. 2019). The cytoskeleton in plants was evolutionally formed under a constant gravity field, and cMTs in particular tend to orient along the mechanical force. Clinorotation does not remove the gravitational force (1 g), but via changes in plant polarity and constant rotation, it distributes gravity load homogenously within the cell. This results in omnilateral hydrostatic pressure produced by the cytoplasm on the cortical cytoskeleton, eventually causing mechanical stress (Ferranti et al. 2021). Many years of investigation still have not provided a clear understanding of how the orientation of the cortical cytoskeleton is controlled and how its connection with PM is regulated. It is known that numerous proteins facilitate the functioning of cMTs by promoting their reorganization, connection to PM and association with AFs (Struk and Dhonukshe 2014; Krtková et al. 2016). We consider some of them to be potential targets of mechanical stimulation (during clinorotation), particularly those involved in the organization and cooperation of cMTs and AFs and their connection with PM. Investigating the proteins that regulate the diverse structural configurations of plant MTs and AFs is important for understanding the general principles of plant growth and development.

Induction of MT polymerization and assembly of cortical arrays are facilitated by MAP65s (MT-Associated Proteins 65), an evolutionarily conserved protein family with a molecular mass of 60–65 kDa (Chang-Jie and Sonobe 1993). In *Arabidopsis,* there are nine members of MAP65s, and some of them display nonoverlapping biochemical functions (Smertenko et al. 2008; Ho et al. 2012). The most studied member of this protein family is MAP65-1, and as all members of the family, it contains a dimerization domain at the N-terminus and a C-terminal MT-binding domain with a spectrin-like fold. Subunits of monomeric MAP65-1 bound to separate MTs form antiparallel dimers and create a cross-bridge between adjacent MTs, promoting their bundling (Smertenko et al. 2008; Subramanian et al. 2010; Tulin et al. 2012).

Cytoplasmic linker protein (CLIP) or cytoplasmic linker associated protein (CLASP) is an MT plus-end tracking protein (+ TIPS class) involved in the regulation of MT plus-end dynamics and stabilization of MT bending at cell edges (Ambrose et al. 2011). It also promotes MTs aligning parallel with maximal stress directions (Uyttewaal et al. 2012). Overexpression of the *CLASP* gene has been shown to stabilize MTs by maintaining polymer status and the formation of extensive oryzalin-resistant MT bundles (Branzzinni et al. 2013). In addition, CLASP is known to link MTs with the endomembrane and regulate auxin transport by interacting with sorting nexin 1 (SNX1), a component of the retromer protein complex responsible for the recycling of the PM auxin efflux carrier PIN2 and promoting in this way plant cell polarity (Kirik et al. 2007; Ambrose et al. 2013). Although CLASP has been found to perform multiple roles (Kirik et al. 2007; Ambrose et al. 2011; Branzzinni et al. 2013), many of its functions in plants remain to be revealed.

Among good candidates for mechanotransduction is plant-specific phospholipase D delta (PLDδ) (Ho et al. 2009; Cvrčkova 2013; Pleskot et al. 2013; Pejchar et al. 2020), a possible mediator in the cell wall–PM-cytoskeleton continuum (Marc et al. 1996; Gardiner et al. 2003) and also able to regulate the activity of both MTs and AFs (Petrasek and Schwarzerova 2009). In particular, PLDδ binds to plant flotillin homolog (Ho et al. 2009), a lipid microdomain marker (Martin et al. 2005), constituting the sites where multimolecular signaling complexes containing G-proteins or kinases, flotillin, PLDδ, MTs, actin filaments, Hsp70 and others are assembled and take part in cell signaling and vesicle trafficking (Martin et al. 2005; Ho et al. 2009; Tapken and Murphy 2015). Moreover, PLD is considered to link MTs and PM, since in *Arabidopsis*, it contains either PH/PX or C2 membrane association domains (Qin and Wang 2002), and some PLDs were shown to be localized at the PM (Liu et al. 2015). Thus, activation of PLDδ results in MT detachment from PM and loss of their parallel order (Dhonukshe et al. 2003), inhibiting normal seedling development (Gardiner et al. 2003). PLDδ may link PM with MTs at sites where cell signaling processes take place (Ho et al. 2009), and PLDδ may play a role in initiating cytoskeleton remodeling.

Formins are proteins that also regulate both MTs and AFs, nucleating and bundling actin and contributing to filament growth, establishing cell polarity, morphogenesis and cell division (Blanchoin and Staiger 2010). AtFH1 (formin-like protein 1) is the most ubiquitously expressed class I formin in *Arabidopsis thaliana*. AtFH1 associates with membranes (Banno and Chua 2000), and its extracellular extensin-like domain may anchor the actin cytoskeleton across the plasmalemma into the cell wall (Martiniere et al. 2011). In addition, AtFH1 was shown to cross-talk AFs and MTs in vivo (Rosero et al. 2013, 2016). Another member of the Class I formins, plant AtFH4, contains a plant-specific transmembrane domain and a specific GOE domain that binds directly to MTs, suggesting a role of AtFH4 at the interface of actin and MT (Deeks et al. 2010). Thus, in plant cells, AtFH4 represents a protein that links membranes and both MTs and AFs (Krtková et al. 2016). Therefore, AtFH4 may transduce mechanical stimuli from the plant cell wall across the PM to both cytoskeletal networks. To some extent, FH4 participates in establishing cell polarity.

Mechanical perturbation achieved by clinorotating plants (Ferranti et al. 2021) is capable of affecting the organization of MTs and AFs in the cortical cell area. It is in agreement with our previous observation of randomized cMTs in the *Beta vulgaris* root transition zone under clinorotation (Shevchenko 1999). Therefore, investigating the gene expression of cytoskeleton-associated proteins under mechanical stimulation might shed light on the mechanism of cytoskeleton regulation, which prompted us to investigate whether clinorotation affects the expression of the *MAP65-1, CLASP, PLDδ, FH1, FH4, TUA6* and *ACT2* genes. To obtain more details on cortical cytoskeleton activity, we performed pharmacological studies with the MT- and AF-depolymerizing drugs oryzalin (ORY) and cytochalasin D (CD), respectively, to slightly disorganize MTs/AFs. This helped us predict how mechanical stimulation affects the connection between cMT/AF organization and the expression of genes coding MT/AF-associated proteins and whether cytoskeleton-regulating proteins are involved in mechanical signal transduction in root cells of *A. thaliana* seedlings.

## Materials and methods

### Plant material and growth conditions

Seeds of *Arabidopsis thaliana* (L.) Heynh (Col-0, WT) were sterilized for 2 min in 70% ethanol and then for 20 min in 5% sodium hypochlorite and 0.02% (v/v) Triton X-100 with constant shaking. After three washes with distilled water, seeds were sown on ½ Murashige-Scoog (MS) medium with agar (0.5%) in plastic flasks or Petri dishes and placed at 4 °C. After 48 h of cold treatment, seeds were placed in the growth chamber (25 °C, 18/6 photoperiod) to start germination for 3–4 days. Nearly sixty 3- to 4-day-old seedlings were transferred each to (1) pure ½ MS medium (control), (2) ½ MS medium with 5 μM ORY; (3) ½ MS with 5 μM CD, (4) ½ MS medium put on clinostats; (5) ½ MS medium with 5 μM ORY put on clinostat, and (6) ½ MS medium with 5 μM CD put on clinostat.

Clinorotation lasted for 2 subsequent days. There was no consistency in our earlier preliminary experiments on gene expression after 6 h of seedling treatment with inhibitors. Perhaps, it was due to the fact that functioning of cortical cytoskeleton has not been stabilized yet after change of gravity load. Moreover, there was no evident difference in root length at earlier stages of development. However, after 1 and 2 days of experiments there was a consistency in gene expression and in response of cytoskeleton elements to the actin/tubulin inhibitors. The cortical cytoskeleton function became adapted to new conditions after almost two days of clinorotation, which was reflected in cytoskeleton related gene expression.

ORY or CD concentrations were finally selected for the analysis based on their relatively moderate inhibitor ability to cause partial disorganization of MT and AF cortical arrays, impair root growth and allow growth renewal after inhibitor removal. Dimethylsulfoxide (DMSO) (Sigma, USA) was used as a solvent to dissolve ORY and CD. Therefore, it was also added to the control MS with the same final concentration of 1% (v/v) as that used in the ORY and CD treatments.

**Fig. 1.**
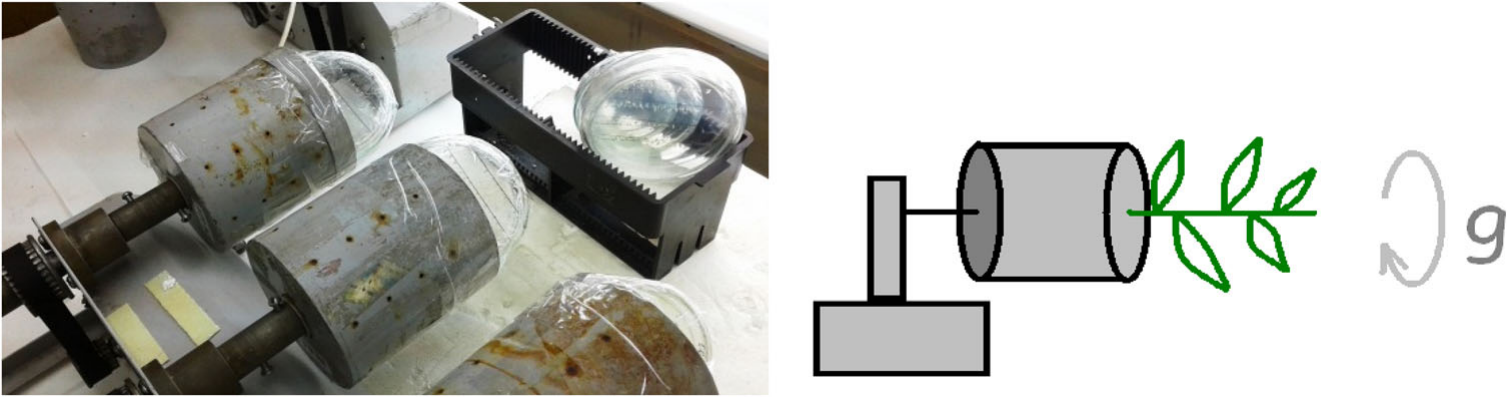
1D clinostat used in the study

For the experiments, a slow rotating (2 rpm) 1D clinostat was used (Fig. 1), where flasks with *A. thaliana* seedlings were rotated horizontally to prevent plants from sensing the gravity vector (Kiss et al. 2019).

Thus, we compared damage to cytoskeleton elements affected by ORY and CD in root cells of seedlings grown in the control (Con) with damage from ORY/CD in clinorotated plants (Clin). In total, there were six sets of treatments: (1) control without any addition (Con), (2) with the addition of ORY or (3) CD, (4) clinorotation without any addition (Clin), (5) clinorotation with the addition of ORY (ClinORY) or (6) CD (ClinCD).

The length of roots was measured before placement on the clinostat and after two days of clinorotation. For RNA isolation, roots of *Arabidopsis* seedlings were dissected from seedlings and collected in 1.5 ml Eppendorf tubes, fixed immediately in liquid nitrogen, and stored at −70 °C for less than a week. For each set of treatments, 50–60 seedlings were processed.

### Statistical analysis of microtubule disorientation and root growth

All experiments were performed in triplicate. The percentage of cells with partially disorganized MTs (deviating by more than 45 degrees from transversal arrangement) was calculated from the total number of cells in the transition root zone. In each replicate nearly 12 roots were analyzed. The difference in such cell types between clinorotated and control seedling roots was significant in all repeats.

The length of seedling roots was measured using the ImageJ program (http://www.imagej.net). The growth inhibition was calculated using the formula *I* = [(*µ*_*c*_ − *µ*_*t*_)/*µ*_*c*_] × 100, where *I* is a percentage of growth rate, *µ*_*c*_—the mean value for root length in the control, and *µ*_*t*_—the mean value for root length under the treatment.

### Microscopy

Observation of the cytoskeleton was performed by using two stably transformed lines of transgenic *A. thaliana* expressing GFP-MAP4 (fusion of the microtubule binding domain of mammalian microtubule-associated protein 4 (MAP4) with the green fluorescent protein (GFP) gene) (Marc et al. 1998) and GFP-ABD (fusion of fimbrin actin-binding domain 2 with GFP gene) (Voigt et al. 2005) fusion proteins, which decorated the full length of MTs and AFs, respectively. *Arabidopsis* lines were kindly provided by Prof. F. Baluška (Institute of Cell and Molecular Botany, Bonn University, Germany). After 2 days of treatment with ORY and CD, cortical root cells at the level of the meristem and transition root zone (as the most sensitive area) were investigated under a confocal microscope LSM 5 PASCAL (Zeiss, Germany) equipped with Plan-Neofluar 40*/0.75 and Plan-Neofluar 100*/1.3 oil lenses. GFP was observed with a 488-nm laser, and fluorescence was collected at 505 nm. Observation of clinorotated seedlings was carried out immediately after removal from the clinostat. Seedlings were placed in MS medium between two parafilm strips on microscopic slides and covered by cover slips that allowed the root to be kept intact. Pictures were exported as graphic files from the LSM Image Browser program installed in the microscope.

### RNA isolation

*Arabidopsis thaliana* seedlings with dissected upper parts (leaf zone) (approximately 80 mg) were grinded with a mortar and pestle with liquid nitrogen, and total RNA was isolated with the RNeasy Plant Mini Kit (Qiagen, Hildesheim, Germany) following the manufacturer’s protocol. RNA quantity was assessed by a spectrophotometer, and RNA integrity was checked by gel electrophoresis. The extracted RNA was treated with DNase (Qiagen, Hildesheim, Germany) to remove potential contamination of genomic DNA. Total RNA (~ 1 µg) was transcribed into cDNA using a reverse transcriptase kit (Thermo Scientific, Vilnius, Lithuania) according to the manufacturer’s instructions.

The expression of genes was analyzed by real-time quantitative PCR (RT-qPCR) performed in an optical 96-well plate using an Analytik Jena's Biometra thermal cycler (Analytik Jena, Jena, Germany) and the following cycling conditions: 15 s at 95 °C, 35 cycles of 1 min at 94 °C, 1 min at 58 °C and 1.5 min at 72 °C, followed by 20 min at 72 °C. Reactions contained SYBR Green Master Mix (Roche Diagnostics Corporation, Indianapolis, USA). Primers for cDNA PCR amplification are presented in Table 1.

**Table 1:** PCR primers for genes encoding microtubule-associated and actin-binding proteins

Gene	NCBI GenBank accession number	PCR primer nucleotide sequence, 5´-3´	
		Forward	Reverse
*ACT2*	AT3G18780	CTTGCACCAAGCAGCATGAA	CCGATCCAGACACTGTACTTCCTT
*TUA6*	AT4G14960	GTTCTGGTTCAGCCTGATGG	CCAGTCCGTACCTCGTCAAT
*MAP65-1*	AT5G55230	ACATTAGTTGCCAAGACCCG	GCCTCCGTTTCTCCTCTTCT
*CLASP*	AT2G20190	CTGTTGAAAGGCTGCATCAA	CGACAACAGCAGGAACAAGA
*PLD*δ	AT4G35790	GGCGGAGAAAGTATCGGAGG	CGAGCTAGAGTCGCTTGAGG
*FH1*	AT3G25500	AGCCAACTTTGAGTCCGAGG	CATCAGCGCCTTTGACATCG
*FH4*	AT1G24150	GAGCTTAGGTCACGTGGCTT	CTTCGGTTAAGCACGCATCG
*SAND*	AT2G28390	AACTCTATGCAGCATTTGATCCACT	TGATTGCATATCTTTATCGCCATC

The specificity of PCR primer pair nucleotide sequences was checked against the *A. thaliana* transcript database using TAIR BLAST (http://www.arabidopsis.org/Blast/) and NCBI BLAST service (http://blast.ncbi.nlm.nih.gov). The PCR-generated amplicons were checked by gel electrophoretic analysis along with a 50-bp DNA-standard ladder (Invitrogen GmbH, Karlsruhe, Germany). The amplicon length for all primers was approximately 90–150 bp. The *SAND* gene was used as an internal DNA standard for normalization. Samples with 1 μl of RNase-free water instead of cDNA were used as negative “no template” controls. Standard curves and primer efficiencies were calculated using qTower2 software (https://www.labwrench.com/thread/206903/analytik-jena-qtower2-software). The efficiency of qPCR was 101% for *TUA6, PLDδ, CLASP* and *FH4* and 103.9% for *ACT2, SAND, FH1* and *MAP65-1* primers. Expression levels for each sample were calculated based on three technical replicates via the standard curve (which takes into account primer efficiency). For each sample, at least three biological replicates were performed (pools of nearly fifty seedlings for each replicate). The 2^−ΔΔCt^ calculation as described in Livak and Schmittgen (2001) was used to determine the relative mRNA levels. The Wilcoxon signed rank test was used to evaluate the difference between each set of measures for each gene. Control and clinorotation treatments were considered to be equal to 1. In the experiments with ORY and CD treatment, gene expression was compared between nonclinorotated and clinorotated samples. Relative gene expression was considered to be downregulated if it differed at least twice or more from the control.

## Results

### Growth test

**Fig. 2:**
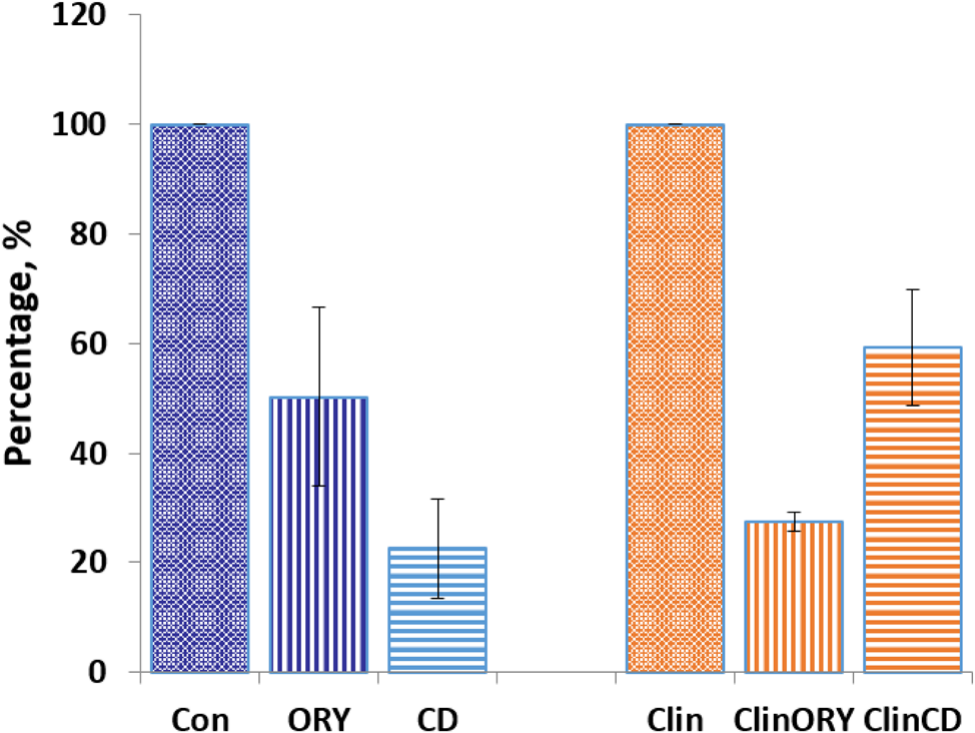
Relative growth rate of *A. thaliana* seedling roots after treatment with oryzalin (ORY) and cytochalasin D (CD), *p* < 0.05; control (Con) and clinorotated (Clin) samples were taken as 100%

The root length of clinorotated *A. thaliana* seedlings was 67.04 ± 8.96% of the roots in the static control. The results of root growth in three replicates are presented in Fig. 2.

The application of ORY and CD caused a more pronounced and statistically significant (*p* < 0.05) decrease in root length in both the control and samples after clinorotation (Fig. 2; Table 2), suggesting that impaired root growth resulted from damaged MTs and AFs. The latter was proved by imaging of cMT and AFs.

**Table 2:** Results of root growth in three replicates

Replicate	*µ*_*c*_	*n*_*1*_	*µ*_*t*_	*n*_*2*_	*t* (*n*_*1*_ + *n*_*2*_* − *2)	*p*
Control vs. Clinostat (set 1 vs. 4)						
1	1.14 ± 0.14	43	0.85 ± 0.15	53	9.55 (93)	1.81E-15
2	1.46 ± 0.25	90	1.01 ± 0.19	77	12.75 (165)	2.34E-26
3	1.25 ± 0.33	67	0.72 ± 0.18	61	9.00 (154)	2.29E-20
Control vs. ORY (set 1 vs. 2)						
1	1.13 ± 0.25	48	0.74 ± 0.16	61	9.93 (107)	7.22E-17
2	1.05 ± 0.18	43	0.78 ± 0.16	53	7.82 (94)	7.54E-12
3	1.53 ± 0.33	42	0.70 ± 0.08	21	11.21 (61)	1.89E-16
Clinorotation without any addition vs. Clinorotation with addition of ORY (set 4 vs. 5)						
1	1.17 ± 0.24	45	0.82 ± 0.19	67	8.66(110)	4.61E-14
2	1.44 ± 0.23	63	0.41 ± 0.09	59	31.56 (120)	0.001
3	1.41 ± 0.28	31	0.67 ± 0.13	31	13.38 (60)	1.14E-19
Control vs. CD (set 1 vs. 3)						
1	1.15 ± 0.38	80	0.47 ± 0.11	63	13.73 (141)	9.21E-28
2	0.58 ± 0.13	69	0.56 ± 0.10	91	1.00 (158)	0.317372
3	1.11 ± 0.22	65	0.58 ± 0.12	49	15.19 (112)	5.77E-29
Clinorotation without any addition vs. Clinorotation with addition of CD (set 4 vs. 6)						
1	0.70 ± 0.22	76	0.48 ± 0.09	45	6.45 (119)	2.45E-09
2	0.66 ± 0.12	88	0.49 ± 0.04	57	10.42 (143)	2.84E-19
3	0.70 ± 0.26	48	0.65 ± 0.18	55	1.17 (101)	0.2453

*µ*_*c*_—mean value for root length in the control, *µ*_*t*_—value for root growth rate under the treatment, *t*—Student's *t*-test value, *n*_1_ and *n*_2_—variance sizes, *p*—statistical probability value

### Organization of cortical tubulin microtubules in root cells of *Arabidopsis* seedlings treated with cytoskeleton inhibitors oryzalin and cytochalasin D and clinorotation

**Fig. 3:**
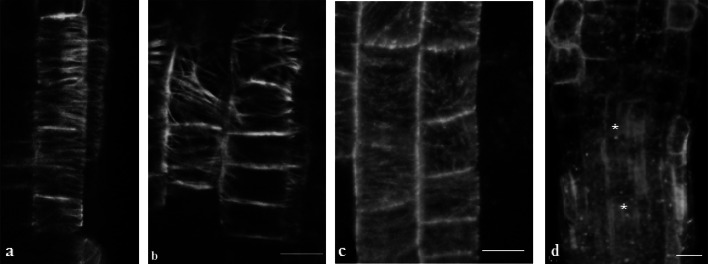
Transverse cMTs in meristem cells of *Arabidopsis* plants without treatment (**a**); cMT deviating from transverse arrangement in cortex cells of clinorotated *Arabidopsis* plants (**b**); partially desorganized cMT in cortex cells of roots treated with ORY (**c**) and CD (**d**) in *A. thaliana-* GFP-MAP4. The cMTs treated with ORY or CD looked similar in *Arabidopsis* plants without clinorotation vs. clinorotated plants. Therefore, photos only for the second ones are presented (**c** and **d**). Snowflakes mark tubulin foci after CD treatment (**d**). Scale bars = 10 μm

In cells of the late meristem and transition zone of *Arabidopsis* plant roots, MTs formed dense arrays, where alignment of separate MTs was preferentially transverse to the longitudinal cell axis (Fig. 3a). The cMT organization in clinorotated samples was slightly different from that in the control plants; in particular, disorganized MTs were revealed in 18.58 ± 2.39% (*p* = 4.77E−12) of cells of the control plant roots and 28.26 ± 2.84% (*p* = 1.62E−08) of the clinorotated roots. Thus, under clinorotation, almost 10% more cells with cMTs notably deviated from transverse organization were observed (Fig. 3b).

After application of ORY, the separate MT arrays were not well pronounced, and cMTs looked blurred in roots of *Arabidopsis* in both static (not presented) and clinorotated plants (Fig. 3c). In cortical root cells, disrupted MT arrays and accumulation of damaged MTs near the cell periphery were observed (Fig. 3c). A distinctive feature of plants treated with CD was the appearance of foci from tubulin located in cells of all root growth zones (Fig. 3d). It should be noted that there were no significant differences regarding cMTs in root cells between static and clinorotated *Arabidopsis-*GFP-MAP4 seedlings treated with ORY or CD, showing the same nature of MT distortions.

### Organization of actin filaments in root cells of *Arabidopsis* seedlings treated with cytoskeleton inhibitors oryzalin and cytochalasin D and clinorotation

**Fig. 4:**
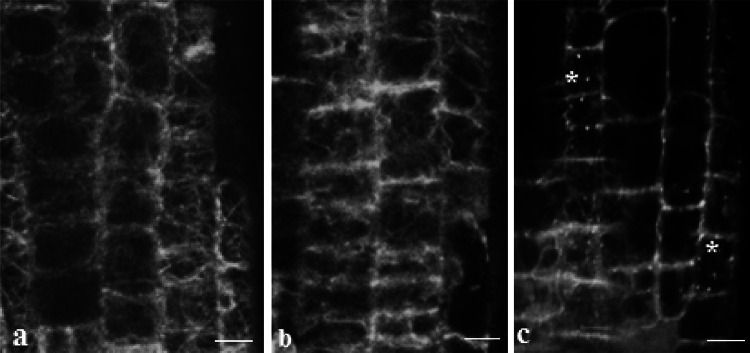
Organization of actin microfilaments (AFs) in static control (**a**), treated with CD (**b**) or ORY (**c**) *A. thaliana*-GFP-ABD2 seedling root cells (level of meristem and transition zone). Snowflakes mark actin foci after ORY treatment (**c**). Scale bar = 10 μm

AFs in epidermal and cortical root cells were represented by interconnected filamentous networks without preferred orientation of separate filaments. Normally, in root cells of *Arabidopsis* seedlings, a dense AF meshwork surrounded the nucleus and vacuoles (Fig. 4a). There was no difference in AF organization between static control and clinorotated *Arabidopsis* plants.

Application of CD caused partial damage to AFs, and in some cells, remnants of AFs were concentrated at the cell periphery, showing denser fluorescence (Fig. 4b). Treatment of *Arabidopsis* roots with ORY resulted in the appearance of foci from actin in cortex cells (Fig. 4c). Visually, the AF network was damaged by ORY/CD to the same extent in both control and clinorotated samples.

### Expression profiles of the *ACT2*, *TUA6*, *MAP65-1*, *CLASP*, *PLDδ*, *FH1* and *FH4* genes in clinorotated plants after oryzalin or cytochalasin D treatment

**Fig. 5:**
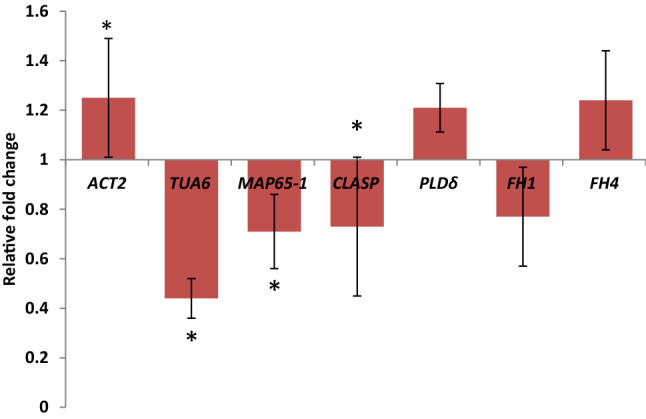
Relative expression of the *ACT2, TUA6, MAP65-1, CLASP, PLDδ, FH1* and *FH4* genes in clinorotated *A. thaliana* seedlings without any inhibitor (set 4) vs. control (equal 1); * *p* < 0.05 based on the non-parametric Wilcoxon test

Analysis of *ACT2, TUA6, MAP65-1, CLASP, PLDδ, FH1* and *FH4* gene expression did not reveal significant changes in *Arabidopsis* seedlings grown on clinostats without any inhibitor (set 4) in comparison to the control (set 1), except for the downregulated *TUA6* and *CLASP* genes (Fig. 5).

**Fig. 6:**
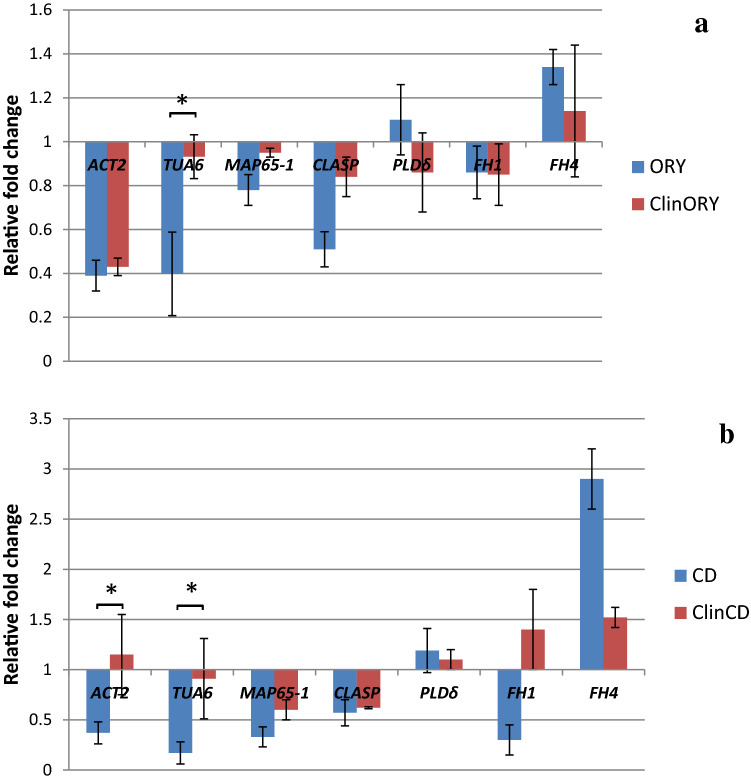
Relative expression of the *ACT2, TUA6, MAP65-1, CLASP, PLDδ, FH1* and *FH4* genes in *A. thaliana* seedlings based on three (*FH1* and *FH4*), five (*ACT2* and *CLASP*), or six (*TUA6*, *MAP65-1* and *PLDδ*) biological repeats with three technical repeats per each biological repeat: **a** - with the addition of oryzalin (ORY, set 2) and the addition of ORY under clinorotation (ClinORY, set 5), *p < 0.05 for ORY vs. ClinORY; **b** - with the addition of cytochalasin D (CD, set 3) and CD under clinorotation (ClinCD, set 6), *p < 0.05 for CD vs. ClinCD; *p* is based on the non-parametric Wilcoxon test

To decipher the mechanism of cytoskeleton regulation under mechanical stress, we performed a pharmacological approach and applied ORY or CD to the growth medium to enhance the effects of damaged MTs and AFs. We investigated the effects of disorganized MTs and AFs on the expression of genes encoding tubulin (TUA6) and tubulin-binding proteins (MAP65-1, CLASP and PLDδ) as well as actin (ACT2) and actin-associated proteins (PLDδ and formins FH1 and FH4), among which PLDδ and FH4 are known to take part in regulating the activity of both MTs and AFs. We paid attention to the interconnection between ORY and CD action on MT/AF organization and *ACT2, TUA6, MAP65-1, CLASP, PLD*δ, *FH1* and *FH4* gene expression and investigated how this interconnection was affected by clinorotation. According to our hypothesis, response to ORY or CD could indicate dependence of *TUA6, ACT2* and *MAP65-1, CLASP, PLDδ*, *FH1* and *FH4* gene expression on the organization of MTs and AFs, respectively. The same treatment upon clinorotation showed that mechanical stimulus affected the dependence of *TUA6, ACT2* and genes encoding cytoskeleton-associated proteins on the organization of MTs and AFs, giving a hint on regulation of the cytoskeleton machinery. Changes in *TUA6, ACT2, MAP65-1, CLASP, PLDδ*, *FH1* and *FH4* gene expression under clinorotation reflected different regulation of associated proteins and possible involvement of the proteins in mechanical stress. The results of our experiments showed that the action of ORY and CD alone downregulated the expression of certain genes encoding cytoskeleton-associated proteins and revealed different modes of AF and MT interconnections in static samples. In particular, comparison of ORY action on *ACT2* gene expression in plants grown without clinorotation and on clinostats showed its downregulation in both cases (Fig. 6a).

This is evidence of an impact caused by disorganized MT on *ACT2* gene expression that suggests an interconnection between MTs and AFs. Since the same impact was observed upon clinorotation, this suggested its independence upon this stimulus (Fig. 6a). The interconnection between cMTs and AFs is evident in images of ORY action on the organization of AFs, which results in partial loss of microfilament network integrity in cortical cells of *Arabidopsis* roots (Fig. 4c).

Seedling treatment with CD affected *ACT2* gene expression in static samples, revealing its dependence on the organization of AFs (Fig. 6b). Imaging also proved partial damage of AFs after application of CD (Fig. 4b). Contrary to ORY, there was a difference in CD action on *ACT2* gene expression in clinorotated plants, and as shown in Fig. 6b, the latter was not changed after AF damage. This might indicate the dependence of *ACT2* gene expression on AF organization during stress produced by clinorotation.

In static plants, treatment with ORY decreased *TUA6* gene expression, while under clinorotation, application of ORY did not significantly change TUA6 (Fig. 6a). This suggests a connection between *TUA6* gene expression and the organization of MTs and shows that this connection is dependent on mechanical stress. Damage of MT organization by ORY led to partial desorganization of the cMT arrays (Fig. 3c). Downregulation of *TUA6* gene expression was observed in the case of plants treated with CD (Fig. 6b). Again, this phenomenon indicates an interrelation between the organization of AFs and *TUA6* gene expression, evidencing mutual dependence of AF and MT organization, which is affected by mechanical stimulation. The organizational connection between MTs and AFs was proven by images of the MT distortion and appearance of tubulin foci in root cells after CD treatment (Fig. 3d).

The application of ORY did not change *MAP-65-1* gene expression either with or without clinorotation, evidencing its independence on the organization of MTs. This also indicates that mechanical stress does not affect the connection of MTs with *MAP65-1*. To our surprise, *MAP65-1* expression was downregulated by CD treatment in nonclinorotated *Arabidopsis* (Fig. 6b), revealing a certain connection between AF organization and *MAP65-1* expression, which was also dependent on clinorotation. To our knowledge, this is the first indication of the connection between AF organization and the expression of the *MAP65-1* gene encoding tubulin-associated protein*.*

Our experiments showed that ORY decreased the expression of the *CLASP* gene in static plants, and it was not affected by ORY in clinorotated plants. The fact that both the *TUA6* and *CLASP* genes were affected by only clinorotation and were not changed in clinorotated plants treated with ORY suggests the dependence of CLASP/MT cooperation on gravity direction (Fig. 6a). Damage to the actin network did not affect *CLASP* gene expression regardless of clinorotation (Fig. 6b).

In our experiments, ORY or CD treatment did not impact *PLD*δ gene expression with or without clinorotation, showing that the organization of MTs and AFs does not regulate the expression of the above gene (Fig. 6). The same was true for the impact of ORY on *FH1* and *FH4* gene expression in static *Arabidopsis* plants.

Downregulation of *FH1* gene expression and upregulation of *FH4* gene expression were noted in static plants treated with CD (Fig. 6b). Regulation of *FH1/FH4* gene expression by CD suggests their connection with the organization of actin microfilaments. CD did not affect *FH1/FH4* gene expression under clinorotation, pointing to the fact that clinorotation differentially regulates the connection between the organization of AFs and *FH1/FH4*.

## Discussion

We chose slow clinorotation in our study to induce a stress response. In cortical cells of the transition zone of plant roots, specific receptors may sense the distribution of PM and mediate the transduction of signals generated by clinorotation, which changes unidirectional impact of gravity and uniformly distributes gravity load within the cell, thus, causing mechanical stress (Ferranti et al. 2021). A crucial role in perceiving the homogenized pressure of clinorotated protoplasts in the cortical area is attributed to the cell wall-PM-cortical MT continuum (Wyatt and Carpita 1993; Baluška et al. 2003). The cortical MT network is connected to the PM via associated proteins (MAPs), and the latter are good candidates for outer signal transduction, including mechanical signals. Actin filaments coalign with cMTs and, therefore, may be involved in signal transduction mediated by associated proteins, which connect AFs to PM and promote cross talk with MTs.

Taking the abovementioned into account, we investigated the expression of the *ACT2, TUA6, CLASP, MAP65-1, PLD*δ, *FH1* and *FH4* genes in clinorotated *A. thaliana* plants treated with either an inhibitor of tubulin polymerization (ORY) or an inhibitor of actin polymerization (CD).

Among all genes tested, the *TUA6* and *CLASP* genes were downregulated by clinorotation alone, which suggests a direct impact of clinorotation on tubulin expression with a subsequent impact on the organization of cMTs. Observation of cMTs deviating from transverse alignment in cells of the *Arabidopsis* transition root zone supports this idea. It should be mentioned that MTs are known to convey a sensory function, and tubulin itself is capable of undergoing spontaneous polymerization due to the dynamic nature of the MT polymer and its capacity for a variety of intracellular and extracellular signals (Murphy and Stearns 1996). The MT polymer can rapidly elongate or shorten (treadmilling) due to the polarity of the heterodimeric protein structure composed of the alpha subunit at the slow growing minus end and beta-tubulin at the fast growing plus end of the polymer (Wade and Hyman 1997). Thus, downregulated *TUA6* and *CLASP* gene expression observed during clinorotation might result in distortion of tubulin polymerization. Referring to CLASP functioning as a stress-dependent MT regulator (together with katanin) (Eng et al. 2021), a decrease in its expression eventually might affect MT stabilization and lead to disorganization of cMT arrays. Nick (2012) even suggested the existence of a specific subpopulation of MTs involved in the perception of mechanical forces. These mechanosensitive MTs promote architectural integration that minimizes mechanical tension produced by gravity. We observed that even minimal distortion of cMT by clinorotation resulted in a decrease in *Arabidopsis* root growth*.* Therefore, it is likely that randomization of cMTs was due to free MT appearing as a result of MT array disorganization. Since the well-known function of CLASP is stabilization of the MT polymer at the growing plus-end, logically, its function is inhibited by distortion of tubulin polymerization. This could happen when plant cells do not perceive gravity to a full extent, and mechanical load is evenly distributed on cortical MTs. It is known that free MTs tend to bind to aligned arrays necessary for establishing a new balance for the cortical plant MT cytoskeleton (Dixit and Cyr 2004; Deinum et al. 2011). Observations of MT severing and reorientation following mechanical stimulation (Uyttewaal et al. 2012) as well as in vitro experiments showing MT stabilization under tension (Hamant et al. 2019) support the abovementioned idea.

### Impact of oryzalin and cytochalasin D on the *ACT2*, *TUA6*, *MAP65-1*, *CLASP*, *PLDδ*, *FH1* and *FH4* genes in static *A. thaliana* seedling roots

Application of actin and tubulin polymerization inhibitors helped to unmask the effects of clinorotation on the organization of cMTs and AFs. This pharmacological approach partially clarified the transcriptional regulation of genes encoding proteins associated with cMTs/AFs. In our experiments, feedback was triggered by inhibition of actin/tubulin polymerization by CD or ORY and downregulation of *ACT2* and *TUA6* gene expression.

It cannot be excluded that the pool of depolymerized actin and tubulin that appeared after CD or ORY action had an inhibitory impact on the expression of their encoding genes. The same impact was noted when the *ACT2* gene was affected by tubulin depolymerization due to ORY, and the *TUA6* gene was affected by CD. This could indicate reciprocal feedback of monomeric tubulin on *ACT2* and distorted and depolymerized actin on *TUA6*, which indicates cross talk between the organization of both MTs and AFs. Functional interconnection between the main cytoskeletal elements was illustrated by our observations of reciprocally distorted MTs/AFs by inhibitors of actin/tubulin polymerization. Remarkably, impact on the cMT organization by CD and damage of the AF network by ORY led to the same results—appearing of small clusters and foci from tubulin and actin, probably due to affection of MT-AF junctions in the cortical cell area. Mutually interdependent cMT-AF coalignment and effects on actin organization by MT-disrupting drugs were shown earlier (Collings 2008; Sampathkumar et al. 2011).

An interesting pattern was observed with the expression of the gene encoding the tubulin-associated protein MAP65-1. In nonclinorotated samples, *MAP65-1* gene expression was not dependent upon cMT damage, indicating that the organization of MTs does not regulate it. This might occur due to the role of MAP65-1 in connecting antiparallel MTs in the cortical cellular network rather than stabilization of the MT polymer. However, to our surprise, *MAP65-1* gene expression was downregulated by actin damage due to CD, implying regulation of *MAP65-1* gene expression by AF organization. To our knowledge, such feedback has not been reported previously. In addition, the connection between *MAP65-1* gene expression and AF organization did not depend on mechanical stimulation. All of the above might reflect a novel function of the MT-binding protein MAP65-1 in the organization of the plant cytoskeleton, in particular, the connection of MAP65-1 with AF organization. Moreover, it opens a bright prospect for further investigations of the role of MAP65-1 in plant cytoskeleton regulation.

Damage of MTs by ORY, downregulated the expression of the *CLASP* gene, another essential player in stabilization of the MT network. It is worth noting the similarity of *TUA6* and *CLASP* gene expression upon damage to MTs by ORY. Analysis showed feedback between MT damage and inhibition of *CLASP* gene expression. Possibly due to MT depolymerization, there is no need for CLASP to cap the MT plus end and prevent tubulin polymer catastrophe (Al-Bassam et al. 2010; Kumar and Wittmann 2012). It is also connected with the ability to align cMTs in parallel with the maximal stress direction, a CLASP function known from experiments on *clasp-1* mutants (Struk and Dhonukshe 2014). However, downregulated *CLASP* gene expression contradicted the increased frequency of rescue events and increased concentration of free tubulin in the presence of CLASP (Al-Bassam et al. 2010; Uyttewaal et al. 2012).

It was documented earlier that formins (plant-specific actin binding proteins) connect PM to MTs and AFs (Deeks et al. 2010) and participate in actin–MT cross-talk (Ho et al. 2009; Deeks et al. 2010; Cvrčkova 2013). In addition, formins can mediate attachment of endomembrane compartments (endoplasmic reticulum or secretory vesicles) to the MT cytoskeleton (Cvrčkova et al. 2015). In our experiments, clinorotation alone did not affect *FH1* and *FH4* gene expression, suggesting no direct impact from the even distribution of gravity load. The application of CD downregulated the *FH1* gene and upregulated the *FH4* gene, which implies the regulation of *FH1/FH4* by the organization of AFs. The opposite effect of CD on the expression of the *FH1* and *FH4* genes means that despite their belonging to the same class, FH1 and FH4 play different roles in the connection of AFs with PM and MTs. Thus, feedback between damaged and depolymerized actin and the expression of the *FH1* gene contribute more to the function of FH1 in stabilizing actin filaments. This is in agreement with reports on the ability of AtFH1 to enhance actin polymerization and AF capping, contributing to overall actin mobility and dynamics (Michelot et al. 2005; Staiger et al. 2009). This phenomenon proves the role of FH1 in the organization of the actin network at the cell cortex and facilitation of cytosolic trafficking (Martiniere et al. 2011). It is worth noting that mutations in *AtFH1* induced actin bundling and, hence, a less dynamic actin cytoskeleton, which in turn caused reduced cell elongation (Rosero et al. 2013; 2016). Damage of AFs by CD makes them less dynamic, which diminishes the role of FH1 in stabilization of the actin network. Thus, a less dynamic AF network results in downregulation of the *FH1* gene. The lack of a connection between *FH1* expression and damaged MT organization could be explained by AtFH1 exclusion from the areas of the cell cortex usually occupied by MTs (Martiniere et al. 2011). AtFH1 is known to anchor actin filaments across the plasmalemma to the cell wall (Martiniere et al. 2011), which may effectively constrain actin bundling, and since FH1 forms a connection between the cell wall and actin cytoskeleton, the effects on its gene expression caused by MT dynamics may be negligible compared to those caused by microfilaments.

Enhancement of *FH4* gene expression during AF depolymerization might indicate its specific role in actin organization, quite different from FH1 activity. Since AtFH4 is considered to interact with both AFs and MTs and be involved in cell signaling, upregulated expression of the *FH4* gene during actin depolymerization definitely points to FH4 as a crucial player in the stabilization of AFs and their connections with MTs and the plasma membrane. In addition, recently, FH4 and its truncated at the C-terminus derivatives that did not have actin binding domains were found to relocate to sites of callose deposition in plant cells attacked by fungal or oomycete pathogens (Sassmann et al. 2018). This indicates functions other than actin-binding of FH4 in the organization and functioning of the cell wall and cytoskeleton. Probably, enhanced expression of the *FH4* gene during AF damage suggests a multidirectional role of FH4 in stabilization of the cell wall–PM-cytoskeleton continuum during stress.

### Impact of oryzalin and cytochalasin D on the *ACT2*, *TUA6*, *MAP65-1*, *CLASP*, *PLDδ*, *FH1* and *FH4* genes in clinorotated *A. thaliana* seedling roots

The possible involvement of cytoskeleton-associated proteins in mechanical signaling is another important conclusion that can be derived from the impact of mechanical perturbation on the regulation of cytoskeleton gene expression. Unchanged expression of *ACT2, MAP65-1* and *FH1/FH4* might be explained by averaged mechanical stress on the cortical cytoskeleton during clinorotation when constantly reverting plant cells do not sense a gravity vector to the same extent as during vertical growth.

In our experiments, ORY treatment downregulated *ACT2* gene expression in both clinorotated and static plants, suggesting independence of *ACT2* gene regulation by MT organization on mechanical stimulation. *ACT2* gene expression was not changed in clinorotated plants treated with CD, contrary to the samples treated with CD only, which suggests different regulation of connection between AF organization and the *ACT2* gene expression during clinorotation. The same was true for the expression of the *TUA6* gene in clinorotated plants not affected by either ORY or CD, which is totally in contrast to ORY/CD-treated static plants (Table 3).

**Table 3:** Differential expression of the *ACT2, TUA6, MAP65-1, CLASP, FH1* and *FH4* genes in static and clinorotated *A. thaliana* seedlings treated with oryzalin or cytochalasin D

Gene	Static		Clinorotated		
	ORY	CD	Clinorotation only	ORY	CD
*ACT2*	+	+		+	
*TUA6*	+	+	+		
*MAP65-1*		+			
*CLASP*	+		+		
*FH1*		+			
*FH4*		+			

+Genes that were differentially expressed compared to the static control

Thus, during mechanical perturbation, there was no feedback between depolymerized tubulin and actin and *TUA6* gene expression or between depolymerized actin and *ACT2* gene expression (Table 3). It could be that regulation of the *ACT2* gene by AF organization and the *TUA6* gene by organization of both cMTs and AFs during mechanical stress induced by clinorotation was performed differently than in static plants treated by ORY/CD. Since during clinorotation the *TUA6* gene expression did not respond to changes of both cMT and AF organization, one may speculate that cMTs are playing a leading role in MT-AF interconnection during mechanical stimulation and promote stable cytoskeleton functioning. However, in general, details of interactions between MTs and AFs in the cortical cell area are still poorly investigated. Sampathkumar et al. (2011) demonstrated that actin filament reassembly depends on MTs with the participation of formin interactions. It has been previously reported that AFs and cMTs interact during the cell response to geometrical perturbation of protoplasts, and the actin network becomes more irregular when MTs are depolymerized. Although Durant-Smet et al. (2020) did not visually observe a dependence of MTs on the actin network, they suggested that actin organization is dependent on the MTs.

At the same time, experiments with *Dictyostelium* cells demonstrated that actin organization undergoes gradual changes during cell reversion after exposure to a change in the direction of a shear stress of 2.1 Pa. At first, actin rapidly disassembled within 60 s after cytoplasm flow reversal and then started to polymerize within 30–60 s in a new place, establishing new cell polarity (Dalous et al. 2008). In animal cells, mechanical forces were documented to cause the formation of a perinuclear actin rim facilitated by nuclear membrane-associated inverted formin-2 (INF2) (Shao et al. 2015). Thus, it cannot be excluded that during clinorotation, the AF network undergoes certain rearrangements not revealed by the current method.

Our experiments did not reveal any changes in *FH4* gene expression after MT damage or action of clinorotation, and this does not suggest a role for the above proteins in mechanosensing. Nevertheless, Deeks et al. (2010) suggested that mechanical stimuli could be transduced by the FH4-mediated PM-cytoskeleton continuum and preferentially can result in changes in actin dynamics. According to the proposed model, MTs act as structural scaffolds that allow the FH2 domain of AtFH4 to perform its actin-nucleating function.

In our experiments, clinorotation did not affect *FH1* and *FH4* gene expression during damage of AF/MTs, which could mean different modes of connection between *FH1* and *FH4* gene expression and the state of AF/MT networks. The same was true for the *CLASP* gene, which was also shown to be regulated differently when MTs were disorganized during clinorotation.

Among the proteins that regulate the dynamics of cMT and are able to participate in mechanical signal transduction is MAP65-1. However, neither clinorotation alone nor application of actin/tubulin inhibitors under clinorotation showed significant changes in *MAP65-1* gene expression. MAP65-1 has a high specificity to form cross-bridges between overlapping antiparallel MTs along the whole length of MTs, suggesting its role in the organization and stabilization of MT arrays (Ho et al. 2009; Tulin et al. 2012). Perhaps, its function in the organization of the cMT network is rather stable and not affected by stimuli such as clinorotation.

In addition, we tested the bifunctional protein PLDδ, which regulates actin–MT ‘crosstalk’ (Petrašek and Schwarzerova 2009). However, despite activation upon salt stress and xylanase or mastoparane treatment (Pleskot et al. 2014), neither clinorotation alone nor inhibitors of MT or AF polymerization induced any changes in *PLDδ* gene expression. On the other hand, MT depolymerization induced by ORY activates PLDα1, and depolymerized G-actin inhibits PLDβ1 activity, indicating feedback from the actin cytoskeleton in the regulation of PLD activity (Pleskot et al. 2014; Zhang and Zhang 2016).

In general, the impact of cMT/AF organization on the expression of genes encoding associated proteins suggests a highly dynamic structure of the plant cytoskeleton and constant remodeling of actin and MT networks regulated by associated proteins.

Finally, although we assume that a change in transcript level of particular genes indicated their functional role in the studied response to clinostat treatment, it should be explicitly stated that (1) absence of transcript level change for some genes would not rule out their functional involvement and (2) observed transcript level change may be quite far downstream of the stimulus and possibly secondary to the impaired growth.

## Conclusions

Thus, our experiments have shown that the organization of cMTs and AFs is able to regulate the expression of the *ACT2, TUA6, MAP65-1, CLASP, FH1* and *FH4* genes. We observed feedback between MTs and AF damage and the expression of the *ACT2* and *TUA6* genes*,* between MT organization and the expression of the *CLASP* gene, and between AF organization and the expression of the *MAP65-1* and *FH1/FH4* genes. Such feedback was not observed during clinorotation, which averaged the impact of gravity and facilitated omnilateral mechanical stimulation of the cortical cell area. For the first time, using transcriptome data, we demonstrated that slow clinorotation is able to cause mechanical stress. We suggest with a high certainty that cMTs play a leading role in plant cell mechanosensing along with MAP65-1, CLASP and formins FH1/FH4. Our study contributes to understanding the fundamental principles of cortical MT and AF organization and their role in the regulation of plant cell growth under stress conditions.
